# A quality improvement project on the discharge summary completion process in an addictions service

**DOI:** 10.1192/bjo.2021.558

**Published:** 2021-06-18

**Authors:** Lily Mohamed

**Affiliations:** Princess of Wales Hospital, Cwm Taf University Health Board, NHS Wales, Leeds & York Partnership NHS Foundation Trust

## Abstract

**Aims:**

Discharge summaries are vital documents that communicate information from hospital to primary care providers. The documents contain description of the patient's diagnostic findings, hospital management, laboratory results, medications list and arrangements for post-discharge follow-up. Ineffective communications between healthcare providers in the form of delayed or poor quality discharge summary may adversely affect patient care and safety.

The setting of this project is Gwent Specialist Substance Misuse Service (GSSMS) which is the statutory specialist addictions service within Aneurin Bevan University Health Board (ABUHB). GSSMS has been arranging and managing inpatient alcohol detoxes for many years. One of the issues highlighted by an inpatient alcohol detox audit in 2017 was discharge summaries were not being completed for every patient who was admitted with a compliance rate of only 57.7%. A quality improvement project was initiated following the presentation of the audit on a Staff Education Day.

The aim of the project is to increase the discharge summary completion rate from 57.7% to 80% by June 2019.

**Method:**

A discharge summary process map was developed to understand the possible causes of delay then Plan, Do, Study, Act (PDSA) methodology was utilised. The result of the original audit was taken as the baseline measurement and benchmarking activities and PDSA cycle were performed. Interventions included root cause analysis by way of brainstorming, education, communication and constructing a checklist.

**Result:**

There has been significant improvement with the compliance rate following the PDSA cycle. It went up to 100% before tapering off to 85% by the end of the project.

**Conclusion:**

Awareness building, continuous monitoring and engagement of teams alongside regular feedback were shown to be the important factors to achieve and sustain the improvement.

